# Obstetrical Auscultation

**Published:** 1852-02

**Authors:** M. M. Rogers

**Affiliations:** Rochester, N. Y.


ART. III.—*Obstetrical Auscultation.* By M. M. ROGERS, M. D., of Rochester, N. Y.

I trust I may be pardoned for the few suggestions I may make on the important subject of obstetrical auscultation. This branch of obstetrics, though by no means new, is still very little studied, and seldom called in requisition in practice. This is more especially the case with practitioners remote from large towns, where the spirit of medical investigation almost necessarily languishes for want of certain facilities to sharpen and stimulate it, which are afforded in cities. Among these means, are hospitals, medical schools, meetings of medical societies, easy access to new publications, and new instruments, opportunities of dissections, and the frequent contact of the profession in consultation, &c.

But fortunately, the branch of science in question, can be studied as well in the country as in the cities; practitioners in the country have more obstetrical practice in proportion, than those in the cities. Obstetrical auscultation is, however, so far as I have observed, neglected to a very great extent, both in town and country. I believe comparatively few of the profession *habitually* call to their aid this means of diagnosis in cases of suspected pregnancy. There are several reasons why this is true:

1. Because treatises on this subject are hardly to be had in this country, except such brief ones as are found in the works on obstetrics. 2. Our knowledge of it is not sufficiently thorough and practical, to cause us to give it the importance due, as a means of diagnosis. 3. Delicacy on the part of physicians as well as patients, in practicing this mode, which nevertheless, is far less repulsive, more delicate and certain in results, than the "*toucher.*"

This method, which is the only one by which a safe and certain diagnosis of pregnancy can be made, is usually the last proposed by the physician, and is considered by most women, to be some "new experiment," to which they submit with distrust and reluctance. This ought not to be; the women and all other patients should be taught what is required of them, and be induced to submit to such reasonable and necessary course as an intelligent physician may propose. But the time to dispense with obstetrical auscultation, or to doubt its utility in diagnosis, and prognosis also, is past,—and those who neglect to avail themselves of its advantages, must be content with second rate success and reputation in this branch. That this is the only means by which we can obtain *positive* evidence of pregnancy, needs no proof.

The facts which may be thus established are briefly:—1. After the fourth or fifth month, whether a fœtus exists in the uterus or not. 2. Whether there are more than one. 3. Whether the fœtus is living. 4. In many cases, whether it is in a state of disease or health. 5. What the presentation will be, before labor commences. 6. Whether there will be a large or small amount of amniotic fluid. 7. After the fifteenth or sixteenth week, whether there be a placenta or mole if no fœtus. These are points on which auscultation gives positive evidence: the absence of the fœtal "*tic tac*" and "*soufflet*" or "*bruit placentaire*," are only negative proof of the contrary of these propositions. The fœtal "tic tac" can be confounded with no other sound; the "bruit placentaire," only with anurismal varix.

Any mode of diagnosis which enables us to establish so many important facts, cannot be unworthy careful investigation.

I believe I hazard nothing in saying, that any man of ordinary acuteness, can acquire a *fair* knowledge of this branch of our profession, by fifty hours study, and ten days practice on the living subject.

In a medico-legal point of view, this knowledge is of vast importance. But I will defer farther remarks for another number of this Journal.

ROCHESTER, January 14, 1852.

